# Redox state and metabolic responses to severe heat stress in lenok *Brachymystax lenok* (Salmonidae)

**DOI:** 10.3389/fmolb.2023.1156310

**Published:** 2023-05-24

**Authors:** Yan Chen, Zhe Pan, Yucen Bai, Shaogang Xu

**Affiliations:** ^1^ National Engineering Research Center for Freshwaters (Beijing), Fisheries Science Institute, Beijing Academy of Agriculture and Forestry Sciences, Beijing, China; ^2^ Ocean College of Hebei Agricultural University, Qinhuangdao, Hebei Province, China; ^3^ China Rural Technology Development Center, Beijing, China

**Keywords:** redox state, lenok, heat stress, metabol/nomics, metabolism

## Abstract

In order to provide new insights into the physiological responses of lenok (*Brachymystax lenok*: Salmonidae) to acute and severe heat stress (25°C, 48 h), dynamic changes in redox state and metabolic responses are studied combined biochemical index and non-targeted metabolome. Nicotinamide adenine dinucleotide (NAD^+^) consumption causes significant increases in ratio of reduced NADH to NAD^+^ and ratio of reduced nicotinamide adenine dinucleotide phosphate (NADPH) to NADP^+^, which induced the redox imbalance in heat stressed lenok. Lowered reduced glutathione/oxidized glutathione (GSH/GSSG) ratios suggested that more oxidized conditions occurred in heat-stressed lenok, leading to membrane lipid oxidation. The first few hours of heat stress promoted the activity of enzymes involved in anaerobic glycolysis (hexokinase, pyruvate kinase, lactic dehydrogenase) and glutamicpyruvic transaminase and glutamic oxaloacetic transaminase, which might lead to consumption of many carbohydrates and amino acid catabolism. These enzyme activities decreased with time in a possible compensatory strategy to manage anabolic and catabolic metabolism, maintaining the redox homeostasis. After 48 h of recovery, NAD^+^, carbohydrate levels and enzyme activities had returned to control levels, whereas many amino acids were consumed for repair and new synthesis. GSH remained at levels lower than controls, and the more oxidized conditions had not recovered, aggravating oxidative damage. Glutamic acid, glutamine, lysine and arginine may play important roles in survival of heat-stressed lenok.

## 1 Introduction

Average global air temperatures are predicted to increase from 4°C to 7°C by the year 2,100, and more frequent and extreme weather events such as transient heat waves and drought are expected (Field, 2014; Brown and Caldeira, 2017). In aquatic ectothermic animals such as fish, water temperature limits enzymatic reactions and affects the stability of molecules responsible for series of biochemical and physiological changes (Barbarossa et al., 2021; Shahjahan et al., 2022). Fish can cope with daily and seasonal variation of environmental temperature through physiological adaptions such as changing metabolism, growth rates, reproduction, and antioxidant responses in natural environments (Li S. et al., 2021; Lopes et al., 2021). However, fish health and survival are threatened in acute heat stress situations, or when the duration of heat stress exceeds their tolerances (Barbarossa et al., 2021).

For fish, increased temperature increases metabolic rates and oxygen consumption (Gilbert et al., 2019; Ern et al., 2020). Acute and severe heat stress cause over production of metabolic by-products such as reactive oxygen species (ROS), which lead to the shift of cellular and extracellular redox equilibria. The redox imbalance can result in irreversible cellular injuries including DNA damage, lipid peroxidation, and protein carbonylation (PC) (Banh et al., 2016; Zhou et al., 2019; Chen et al., 2023). These irreversible oxidative modifications compromise biological functions, and consequently induce development of pathologies. Therefore, fish subjected to acute heat stress must activate and coordinate catabolic and anabolic pathways, and simultaneously manage changes in redox state (Skjærven et al., 2013).

The redox state indicates the state of equilibrium between oxidation and reducing capacity, referring to the ability of a reducing substance to transfer electrons to an oxidizing substance in a redox reaction, and the ability of a substrate to gain or lose electrons (Skjærven et al., 2013; Nakano et al., 2014). As oxygen consumption increases with increased temperature, the extracellular redox state may change because of overproduction of ROS (Zhou et al., 2019). The redox state is strictly controlled by regulation of concentrations and ratios of several redox couples, of which the most important are the glutathione couple (reduced glutathione (GSH)/oxidized glutathione (GSSG), and the coenzymes of nicotinamide adenine dinucleotide (NADH/NAD^+^) and nicotinamide adenine dinucleotide phosphate (NADPH/NADP^+^) (Williams et al., 2020; Chen et al., 2022a). Effects of acute and severe heat stress on redox state based on GSH and GSSG levels in salmon were reported by Toshiki (2014). Unfortunately, studies on the acute heat stress responses of fishes have focused primarily on physiological responses or antioxidant defenses. Little is known of responses in the redox state to acute and severe heat stress situations.

Heat stress affects metabolic rate by limiting enzymatic reactions at a cellular level. Fish metabolic responses may vary depending on the magnitude of heat stress. Biomarkers and system biology methods have been applied to analyze metabolic modulations in cold-water fish, including metabolic rates, energy metabolism, and expression levels of metabolites induced by heat stress (Souza et al., 2018; Yang et al., 2020; Li S. et al., 2022; Zhao et al., 2022). Li P. et al., 2021 reported transcriptomic responses of the AMP-activated protein kinase signaling pathway and phenylalanine metabolism in *B. lenok tsinlingensis* exposed to acute and severe heat stress. Liu et al. (2019) investigated global metabolic changes by combining an NMR-based metabolomic strategy and high throughput RNA-Seq, and suggested glutamate as a biomarker in *B. lenok* to indicate the occurrence of sub-lethal high temperatures. Acute and severe heat stress responses may cause unrecoverable metabolic disorders and decrease disease resistance in cold-water fish.

Lenok (*B. lenok*), a land-locked salmonid that frequents upstream regions of cold rivers in East Asia, is endangered in Korea and China (Liu et al., 2019; Chen et al., 2022b). Recently, large-scale artificial reproduction of the lenok have been carried out for the purpose of conservation and commercialization of this species. Normal water temperatures for this species are between 6°C and 18°C. Previous studies have demonstrated that heat shock, and metabolic modulation and immune responses of lenok in heat stress situations change depending on the magnitude and duration of thermal exposure (Liu et al., 2019; Li P. et al., 2021; Chen et al., 2022b). Tolerance to extreme events, such as high temperatures and droughts, may affect their geographic distribution. In the present study, we investigated acute and lethal heat-stress-induced dynamic changes in redox state and metabolic responses, and assessed the recovery patterns after a 48 h of recovery without heat stress. Additionally, we compared the physiological responses of first nine lenok that showed loss of equilibrium (heat sensitive) with those that survived after 48 h of heat stress (heat tolerant) to identify differences between heat-sensitive and heat-tolerant individuals. Global metabolic responses of these heat-sensitive and heat-tolerant lenok, as well as those sampled after 48 h of recovery were investigated by UPLC-MS/MS. These results provided new insights into the physiological responses of lenok to acute and severe heat stress.

## 2 Materials and methods

Animal experimentation was approved by the Institutional Animal Care and Use Committee of the Beijing Academy of Agriculture and Forestry Sciences, Beijing, China. Lenok (average weight 31.2 ± 1.6 g) were obtained from a local hatchery. A total of 210 healthy lenok were divided equally into three fiberglass tanks in the laboratory (70 fish in each), and acclimated at ambient temperature (14°C ± 0.5°C) in a 12-h light/12-h dark photoperiod for 2 weeks in water of pH 7.75, with dissolved oxygen maintained above 7.5 mg L^−1^ through continuous aeration. Fish were fed commercial formula at 3% their body weight twice daily. Half of the water in each tank was changed daily.

Preliminary experiment have been conducted to determine the heating rate and lethal temperature. Based on the lethally rates, we selected 25°C (at a heating rate of 1°C h^−1^) as the experimental heat-stress temperature, at which it killed most of the lenok within 48 h. The 48 h were long enough to distinguish the heat-sensitive and heat-tolerant individuals (Li P. et al., 2021). The water temperature in each tank was incrementally increased by 1°C h^−1^ until 25°C was reached, after which temperature was maintained (±0.5°C) for 48 h. During heat stress, dead fish were removed immediately.

At the start of the experiment, nine fish (3 fish from each tank) were randomly sampled from the 14°C ± 0.5°C water temperature and separated as the control group (C). Because temperature was increased by 1°C h^−1^, heat stress might have commenced at any point in the 11 h during which water temperature was incrementally increased from the ambient (and control) 14°C. We define the time at which water temperature reached 25°C to be ‘0 h’ in terms of heat stress. There were nine fish were randomly selected from the three tanks (3 fish from each tank) and the liver samples were collected at 0, 12, 24, 36, and 48 h, respectively.

The first 9 fish to lose of equilibrium were regarded as sensitive to heat stress (S-group fish), and liver samples were collected from them. The fish that survived after 48 h of heat stress were treated as tolerant to heat stress (T-group fish), and 9 liver samples were collected. The remaining survival fish were transported to a tank containing water at ambient temperature (14°C ± 0.5°C) and allowed to recover for 48 h (R-group fish), and 9 liver samples were collected. Fish were euthanized with MS-222 (∼250 mg L^−1^) before liver sample collection, and liver samples were placed into liquid nitrogen and stored in a −80°C refrigerator prior to subsequent analyses.

### 2.1 Biochemistry

Weighed liver samples were added to pre-cooled normal saline in a weight (g) to volume (mL) ratio of 1:10, and centrifuged at 2,500 rpm for 10 min. The supernatant was collected to determine total protein, NAD^+^/NADH, NADP^+^/NADPH, and GSSG/GSH redox couples, the activities of metabolic enzymes including hexokinase (HK), pyruvate kinase (PK), lactic dehydrogenase (LDH), glutamic-pyruvic transaminase (ALT), and glutamic oxaloacetic transaminase (AST). All commercial assay kits were sourced from Nanjing Jiancheng Bioengineering Institute, China.

Total protein was measured using the Coomassie brilliant blue staining method. NAD^+^ and NADH were extracted using acid and alkaline solutions, respectively. NAD^+^ can be reduced to NADH by alcohol dehydrogenase. Thiazolyl blue tetrazolium bromide (MTT) is reduced to formazan by NADH, through the role of the hydrogen PMS, generating a product absorbs light at 570 nm. Detection reagents were sequentially added according to commercial enzyme kit A114-1-1 instructions. NADP^+^ and NADPH were extracted by acid and alkaline solutions, respectively. NADP^+^ was reduced by glucose 6 phosphate into NADPH. MTT is reduced to formazan by NADPH through the role of the hydrogen PMS, generating a product that absorbs light at 570 nm. Detection reagents were sequentially added in accordance with commercial enzyme kit A115-1-1’s instructions. TGSH and GSSG contents were measured according to commercial enzyme kit A061-2-1’s instructions, based on protein precipitation and the subsequent reaction of non-protein thiols with Ellman’s reagent, generating a product that absorbs light at 412 nm. GSH content represents the difference between TGSH and GSSG.

Hexokinase activity was determined using hexokinase assay kit A077-3-1 specifically, and the reduction of NADP^+^ was monitored at 340 nm. PK activity was determined by PK assay kit A076-1-1, with PK catalyzing phosphoenolpyruvic acid to produce pyruvate, which was then converted to lactic acid by LDH, along with conversion of NADH into NAD^+^. LDH activities were determined using commercial kit A020-1-2, based on decreased absorbance at 340 nm resulting from oxidation of NADH present in the reaction system.

ALT activities were determined by commercial assay kit C009-1-1 (Reitman–Frankel method). Briefly, ALT catalyzes alanine and α-ketoglutaric acids to produce pyruvate and glutamic acid (37°C, pH 7.4). After 30 min, 2, 4-dinitrophenylhydrazide (DNPH) solution was added to stop the reaction, and phenylhydrazone pyruvate (color reaction, 505 nm) was produced at the same time. AST activity levels were determined according to AST/GOT assay kit C010-1-1, where AST catalyzes the translation of amino and ketone groups between glutaric and aspartic acids to produce glutamic and oxaloacetic acids. Oxaloacetate can decarboxylate itself into pyruvate, which reacts with DNPH, with color absorbance measured at 505 nm.

Weighed liver tissue was added into the lysis buffer in a weight (g): volume (mL) ratio of 1:10, and treated in a high-speed grinder. Reagents were added according to electron transport chain Complex I assay kit A089-1-1’s instructions. One unit of activity of NADH dehydrogenase is defined as the amount of enzyme required to catalyze 1 μmol of NADH into NAD^+^ min^−1^.

### 2.2 Metabolite extraction and detection

Weighed liver samples were added into a pre-cooled methanol/acetonitrile/water solution in a 2:2:1 volume ratio, followed by vortex mixing, ultrasound at low temperature for 30 min, and standing at −20°C for 10 min. The supernatant was vacuum dried. Then 100 μL acetonitrile solution was added for resolution, and the solution again was vortexed and centrifuged at 14,000 × g for 15 min. The supernatant was collected for mass spectrometry.

Samples were injected by automatic sampler and separated using an Agilent 1,290 Infinity LC ultra-high performance liquid chromatography (UHPLC, Agilent, United States) HILIC column (25°C, 0.5 mL min^−1^). Quality control (QC) samples were inserted into the sample queue to monitor and evaluate system stability and experimental data reliability. Mass spectrometry was performed on a Triple TOF 6600 mass spectrometer (AB SCIEX). Positive and negative ion modes of electrospray ionization were used for detection. The MS parameters were set as follows: ion source gas 60 psi; ion source gas 2, 60 psi; curtain gas, 30 psi; source temperature, 600 °C; ion spray voltage, ± 5500 V for negative mode and positive mode; mass scan range, 60–1,000 Da for MS and 25–1,000 Da for MS/MS; declustering potential (DP), ± 60 V; collision energy, 35 ± 15 eV; secondary mass spectra were obtained using data dependent acquisition mode (IDA), 4 Da for dynamic exclusion range of isotope ions and 10 fragment maps per scan.

Raw data were converted into mzXML format using ProteoWizard, followed by XCMS software for peak alignment, retention time correction, and extraction of peak area. Metabolite structure identification, and data pre-processing and evaluation of experimental data quality were performed before data analysis. The total ion chromatogram of the QC sample was chromatogram overlapped and compared, and QC samples were analyzed by PCA to evaluate replicate quality. Metabolites extracted from samples were identified by matching with retention time, molecular mass, second-order fragmentation spectrum, collision energy, and other information in-house database (Shanghai Applied Protein Technology). All metabolites identified in both positive and negative ion modes were classified according to their chemical taxonomy.

### 2.3 Statistical analysis

Data were analyzed using SPSS 18.0 (Chicago, SPSS Inc.) and are expressed as means ± standard deviation (S.D.). After checking for normality and homogeneity of variance, redox couples, metabolic enzyme activities, and ATP were evaluated by one-way analysis of variance (ANOVA) followed by LSD multiple analyses to identify any significant differences (*p* < 0.05) between time intervals.

After sum-normalization, processed data were subjected to multivariate analysis in the R package “ropls,” including Pareto-scaled principal component analysis (PCA), and orthogonal partial least-squares discriminant analysis (OPLS-DA). Model robustness was evaluated by permutation testing. The variable importance in the projection (VIP) value of each variable in the OPLS-DA model was calculated to indicate its contribution to the classification. Student’s t tests were applied to determine the significance of differences between two groups of independent samples. Differential metabolites were identified by VIP values (VIP > 1) in the OPLS-DA model, and Student’s t-test (*p* < 0.05).

Hierarchical cluster analysis was performed to display relationships between samples, and differences in metabolite expression patterns. Differential metabolites in each group (compared with control fishes) were mapped to the Kyoto Encyclopedia of Genes and Genomes (KEGG) identifier to implement a pathway analysis. Significant pathways (*p* < 0.05) were selected using KEGG.

## 3 Results

During the heating period, there were no fish showed loss of equilibrium until reach to the target temperature. The lenok show signs of stress including faster swimming and increased opening and closing of operculum at the first hours of heat stress. The first nine fish showed loss of equilibrium were collected from 0 to 12 h after heat stress, which were regarded as the heat sensitive group. With prolonged heat stress, the lenok showed slower swimming and lags in response. After 48 h of heat stress, there were 23 surviving lenok were placed into recovery tanks.

### 3.1 Effect of heat stress on redox couples

**FIGURE 1:**
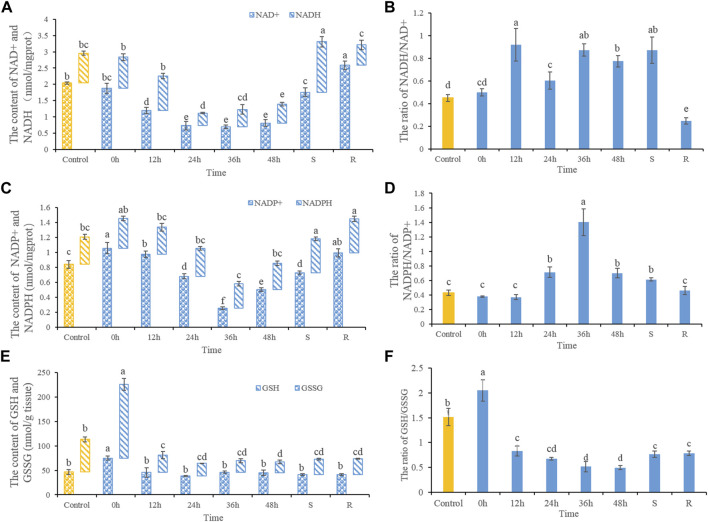
The dynamic changes of the contents of NAD^+^ and NADH **(A)**, the ratios of NADH/NAD^+^ **(B)**, NADP^+^ and NADPH **(C)**, NADPH/NADP^+^ **(D)**, GSSG and GSH **(E)** and the ratios of GSH/GSSG **(F)**. Different letters indicate significant differences between treatments (*p* < 0.05). The same letters indicate no difference between treatments (*p* > 0.05).

NAD^+^ and NADH contents decreased after heat stress (Figure 1A). NAD^+^ contents decreased significantly after 12, 24, 36, and 48 h of heat stress, as well as in S-group fishes. NAD^+^ contents increased significantly after 48 h of recovery when compared with controls. While ratios of NADH/NAD^+^ increased significantly after heat stress. After 48 h of recovery, the NADH/NAD^+^ ratio decreased significantly (Figure 1B). NADP^+^ contents increased after 0 and 12 h heat stress, but decreased significantly after 36 and 48 h heat stress (Figure 1C). After 48 h of recovery, NADP^+^ contents trended upwards, but remained lower than control values. NADP^+^ contents in S-group fishes increased significantly compared with control. The NADPH/NADP^+^ ratio increased significantly after 24, 36, and 48 h of heat stress, and in S-group fishes. Ratios of NADPH/NADP^+^ had recovered after 48 h (Figure 1D). GSSG contents showed increases in 0 h heat-stressed fish, but did not significantly differ otherwise. GSH contents increased significantly in 0 h heat-stressed fish, but then decreased after 12, 24, 36, and 48 h of heat stress, as well as in S-group fishes. GSH levels had not recovered after 48 h (Figure 1E). Ratios of GSH/GSSH increased significantly in fishes at 0 h of heat stress. Ratios of GSH/GSSH decreased significantly after 12, 24, 36, and 48 h of heat stress, as well as S- and R-group fishes when compared with the control (Figure 1F).

### 3.2 Effect of heat stress on metabolic enzyme activity

**FIGURE 2:**
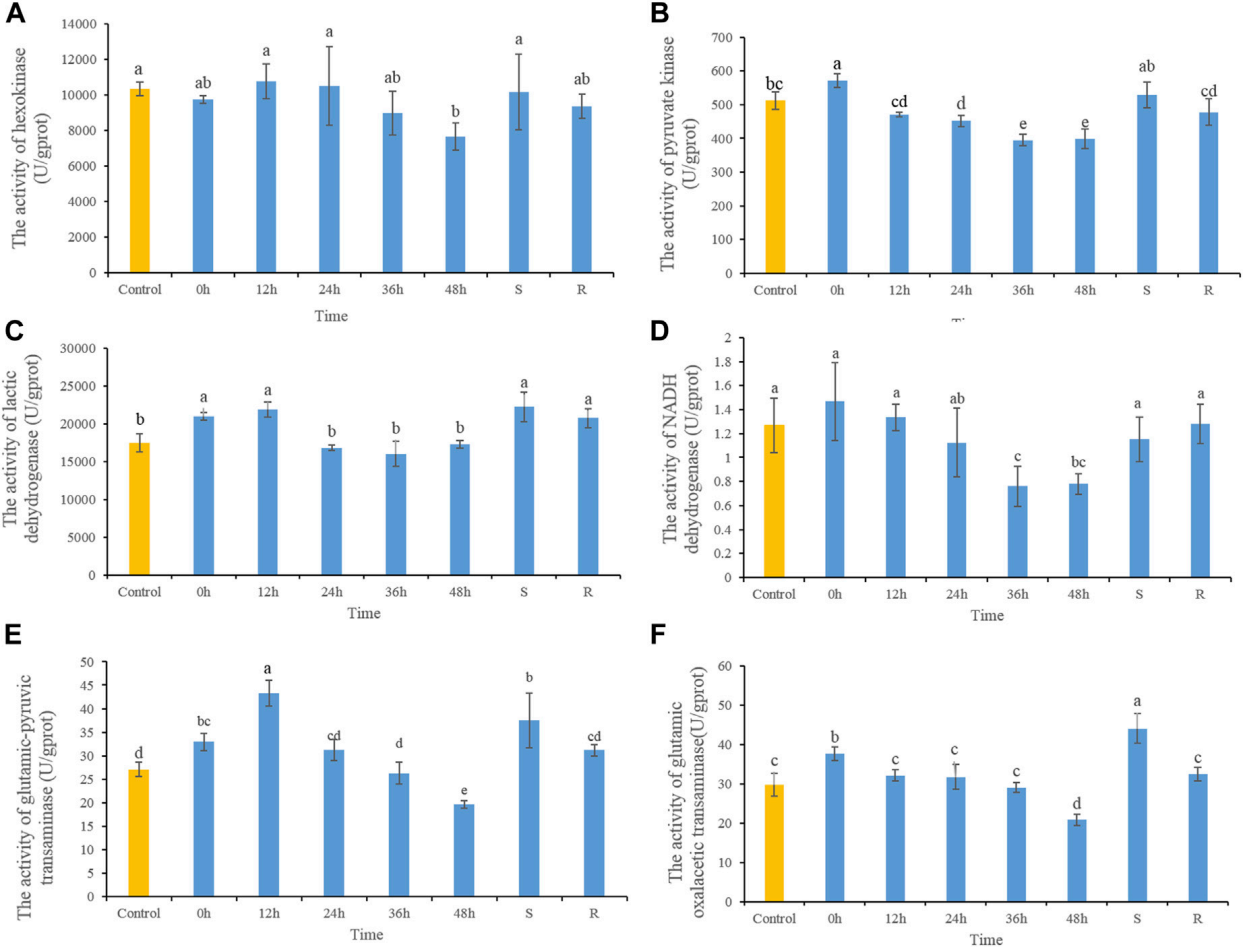
The dynamic changes of the contents of HK **(A)**, PK **(B)**, LDH **(C)**, NADH dehydrogenase **(D)**, ALT **(E)** and AST **(F)** in lenok after acute and severe heat stress and the recovery pattern. Different letters indicate significant differences between treatments (*p* < 0.05). The same letters indicate no difference between treatments (*p* > 0.05).

The HK activity decreased significantly after 48 h heat stress, while there were no significant differences at any other time, or in S- or R-group fishes (Figure 2A). After heat stress, PK activity increased significantly at 0 h, and then decreased gradually. PK activities decreased significantly in 24, 36, and 48 h, and after 48 h of recovery had returned to control. PK activity in S-group fishes did not differ significantly from those in the control group (Figure 2B). LDH activity increased significantly at 0 h, and then decreased gradually which showed significant decreases after 36 h. LDH activity of S- and R-group fishes did not differ significantly from control fishes (Figure 2C). NADH dehydrogenase activity decreased significantly after 36 and 48 h heat stress, but did not differ significantly from control fishes in S- and R-group fishes (Figure 2D). After heat stress, ALT activity increased significantly at 0 and 12 h, and then decreased gradually and showed significant decreases at 48 h. After 48 h of recovery, ALT activity did not differ significantly from control-group fish values. ALT activity in S-group fishes was significantly higher than control values (Figure 2E). Variation in AST activity was similar to that of ALT, which demonstrated increasing significantly at 0 h and then decreasing significantly at 48 h. After 48 h of recovery, AST activity was comparable to control values. AST activity of S-group fishes was significantly higher than control values (Figure 2F).

### 3.3 Effect of heat stress on metabolic enzyme activities

A total of 1702 metabolites were identified in positive and negative ion mode, including 456 lipids and lipid-like molecules, 352 organic acids and derivatives, 178 organoheterocyclic compounds, 143 organic oxygen compounds, 134 benzenoids, 81 phenylpropanoids and polyketides, 72 nucleosides, nucleotides, and analogues, 42 organic nitrogen compounds, 6 alkaloids and derivatives, 4 lignans, neolignans and related compounds, 1 organometallic compound, 1 organosulfur compound, and 232 that were undefined.

**FIGURE 3:**
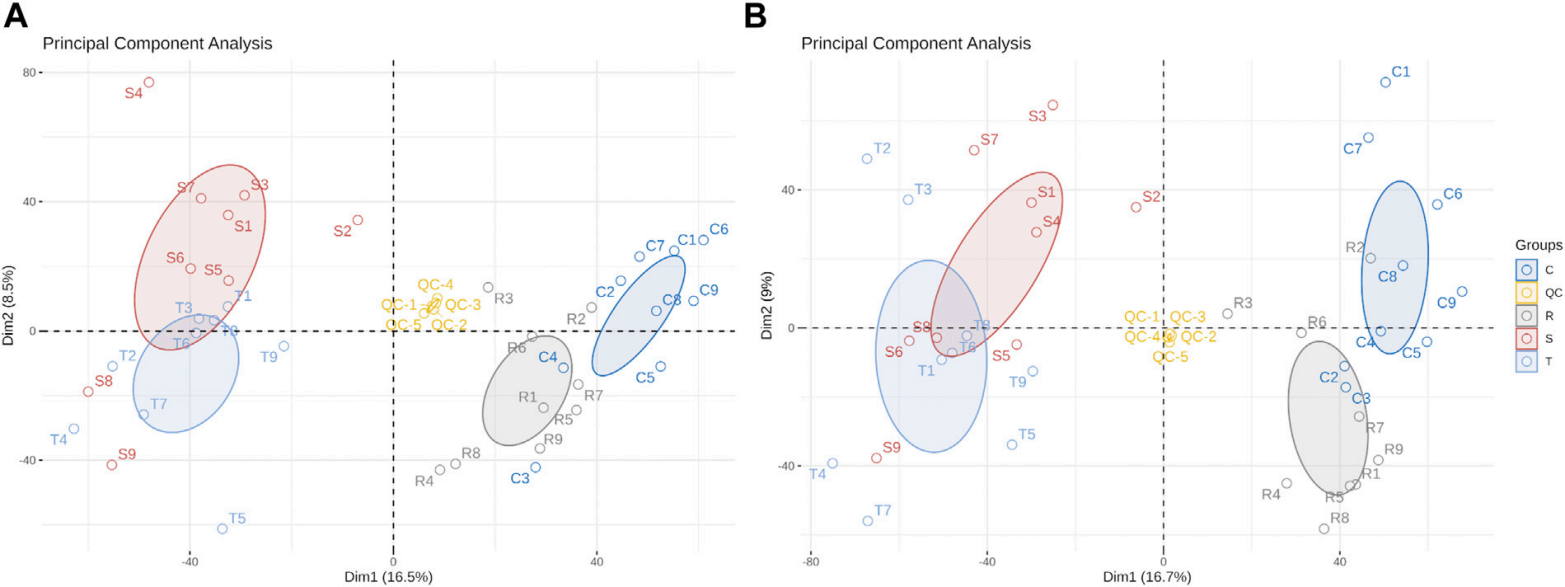
Principal component analysis scores scatter plot of 36 lenok liver samples and QC samples. **(A)** PCA score scatter plot of test samples and quality control samples in positive ion mode. **(B)** PCA score scatter plot of test samples and quality control samples in negative ion mode. C, control group; S, S-group fish; T, T-group fish; R, R-group fish; QC, quality control samples.

Differences in metabolites were distinguished by multivariate statistical analysis, including PCA and OPLS-DA. QC samples clustered close together under positive and negative ion modes, indicating that the instrument had high stability and reproducibility (Figure 3). Model quality is estimated by R^2^X or R^2^Y and Q^2^ values. For PCA, R^2^X = 0.512 in the positive ion mode and 0.518 in the model of negative mode (with QC), indicating that the PCA model had good stability.

OPLS-DA modes which can reduce system noise and extract variable information were applied to further analyze differences between treatment and control groups. Furthermore, 200 permutation tests were used to the over-fitting verification on the OPLS-DA model. For control- and S-group fishes, R^2^Y = 0.984 and Q^2^ = 0.928 in the positive ion mode, and 0.972 and 0.877, respectively, in the negative ion mode (Supplementary Figure S1). For control- and T-group fishes, R^2^X = 0.387, R^2^Y = 0.99 and Q^2^ = 0.947 in positive ion mode, and 0.593, 0.99 and 0.932, respectively, in negative ion mode (Supplementary Figure S2).For control- and R-group fishes, R^2^Y = 0.99 and Q^2^ = 0.863 by permutation test in positive ion mode, and 0.99 and 0.841, respectively, in negative ion mode (Supplementary Figure S3). Values indicate that the OPLS-DA model is highly predictable and suitable for subsequent data analysis in each group. Lower Q^2^ values in permutation tests indicate that the OPLS-DA model is reliable and valid.

**FIGURE 4:**
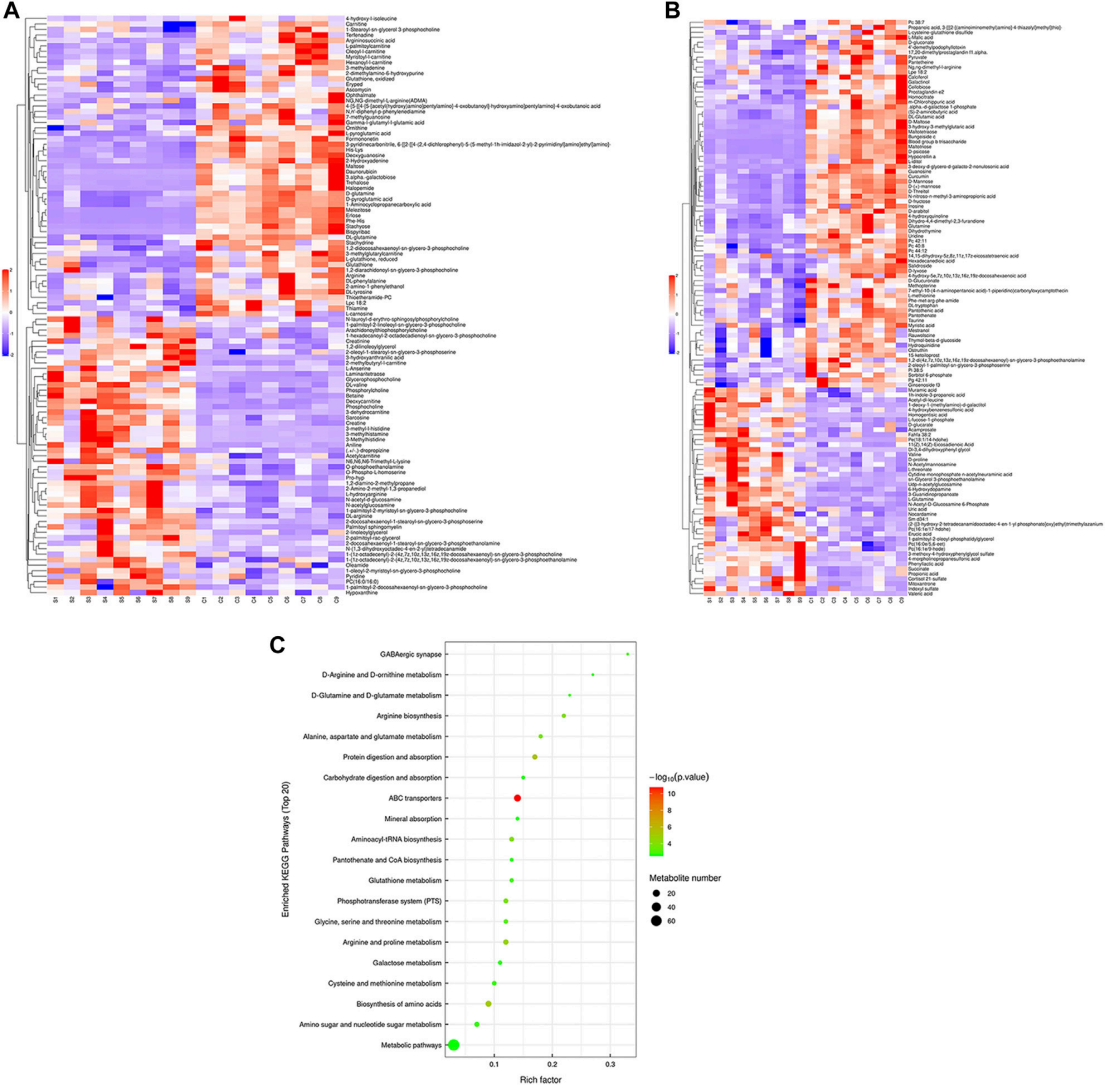
Heat-map visualization of the differential metabolites (VIP > 1, *p* < 0.05) in S-group fish in positive mode **(A)** and negative mode **(B)**, respectively. Blue and red dots indicate the up- and downregulated metabolites. **(C)** Bubble diagram of the TOP 20 significantly enriched KEGG pathways of the discrepant metabolites through the use of KEGG database. Bubble color indicates the degree of significance from the highest (red) to the lowest (green).

**FIGURE 5:**
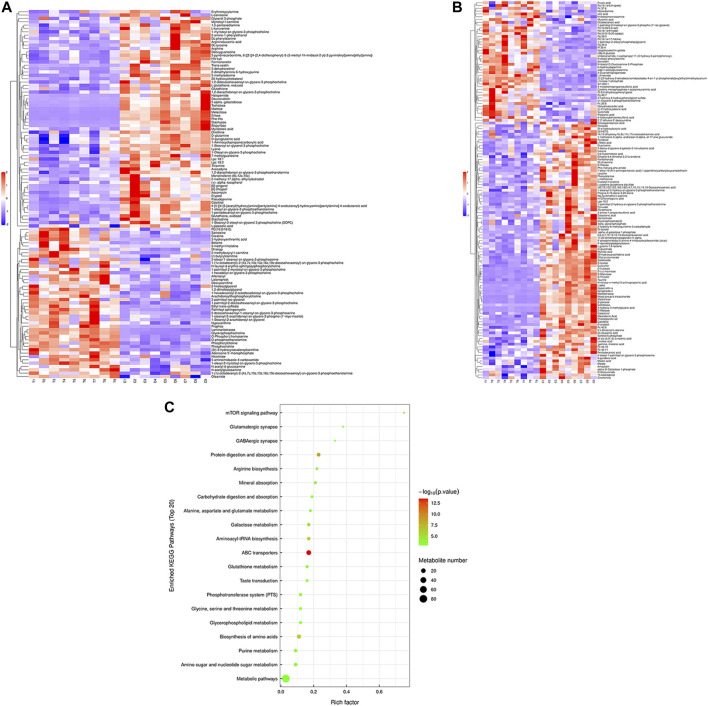
Heat-map visualization of the differential metabolites (VIP > 1, *p* < 0.05) in T-group fish in positive mode **(A)** and negative mode **(B)**, respectively. Blue and red dots indicate the up- and downregulated metabolites. **(C)** Bubble diagram of the TOP 20 significantly enriched KEGG pathways of the discrepant metabolites through the use of KEGG database. Bubble color indicates the degree of significance from the highest (red) to the lowest (green).

**FIGURE 6:**
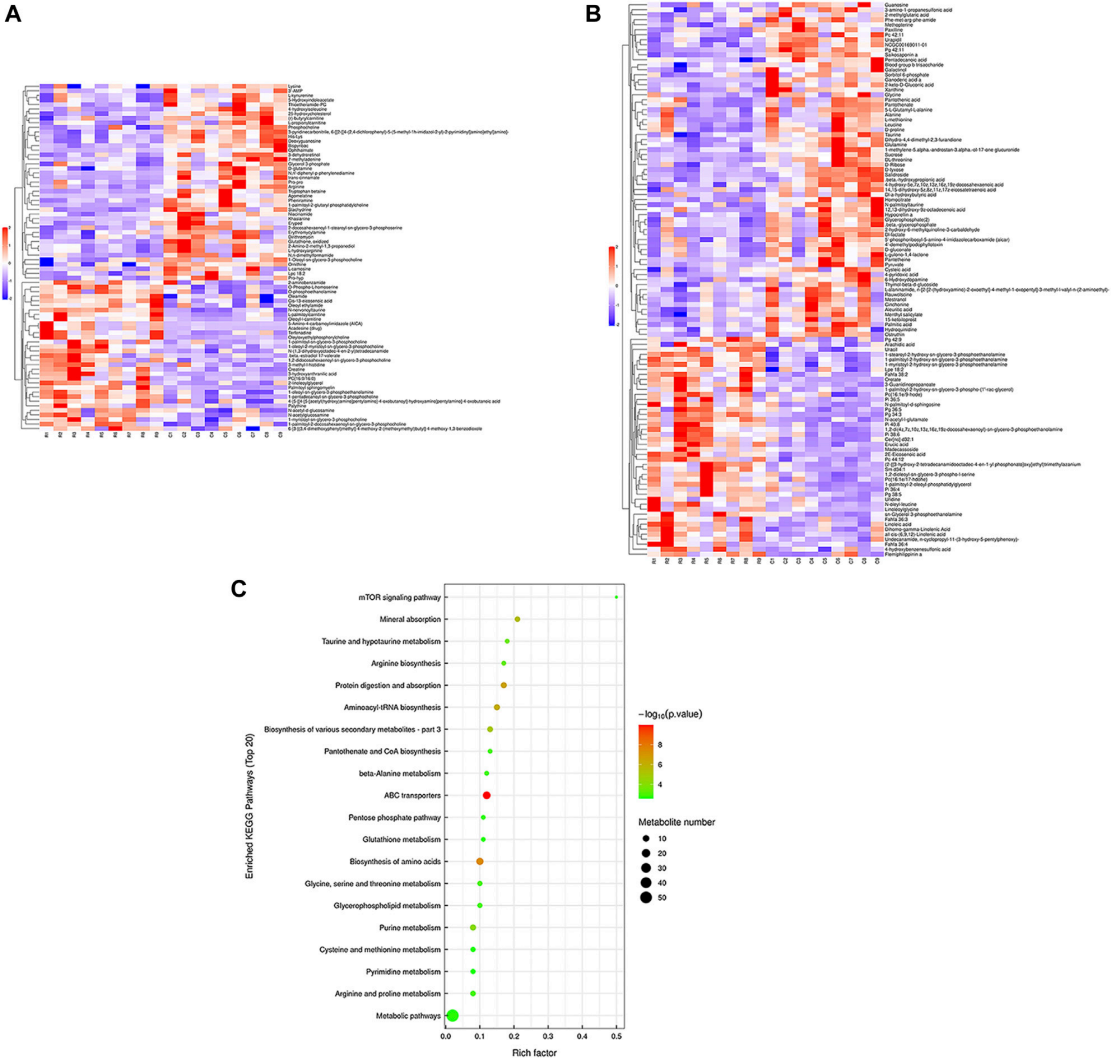
Heat-map visualization of the differential metabolites (VIP > 1, *p* < 0.05) in R-group fish in positive mode **(A)** and negative mode **(B)**, respectively. Blue and red dots indicate the up- and downregulated metabolites. **(C)** Bubble diagram of the TOP 20 significantly enriched KEGG pathways of the discrepant metabolites through the use of KEGG database. Bubble color indicates the degree of significance from the highest (red) to the lowest (green).

Hierarchical cluster analysis was performed to represent the relative content and relationships between differential metabolites. Heat maps are used to present significantly differentially expressed metabolites. Of 106 metabolites in S-group fishes (Figure 4A), 110 metabolites in T-group fishes (Figure 5A), and 76 metabolites in R-group fishes (Figure 6A) changed significantly in positive ion mode compared with control-group fishes. In the negative mode, 116 metabolites in S-group fishes (Figure 4B), 126 metabolites in T-group fishes (Figure 5B), and 111 metabolites in R-group fishes (Figure 6B) changed significantly compared with control-group fishes. Details of metabolites that differed significantly in each group are listed in the Supplementary Table.

To further explore potential metabolic pathways affected by heat stress and recovery, differential metabolites were used as objects to search against the KEGG pathway database (KEGG, https://www.kegg.jp/). Compared with control-group fishes, the top 20 significantly enriched (*p* < 0.05) pathways in groups S, T, and R are shown in Figure 4C, Figure 5C, and Figure 6C, respectively.

## 4 Discussion

More frequent, extreme thermal events are expected with changing climates that will likely affect populations of cold-water fishes such as lenok (Liu et al., 2019; Li P. et al., 2021; Chen et al., 2022b). Molecule stability and enzymatic reactions in poikilothermic fishes are affected by temperature, and any increase in it will promote routine metabolic rates and oxygen consumption. For fish to survive acute and severe heat stress they must activate and coordinate catabolic and anabolic pathways, and simultaneously manage the redox state imbalance and metabolic by-products (Schafer and Buettner, 2001; Skjærven et al., 2013).

NAD(P)^+^ and its reduced form NAD(P)H play key roles in hydrogen transfer in biochemical reactions, which are essential cofactors in redox metabolism (Katsyuba et al., 2020). Ratios of NADH/NAD^+^ and NADPH/NADP^+^ increased significantly, and significant changes in ratios of redox couples indicate that acute and severe heat stress induce a redox imbalance in lenok. Both NADH and NAD^+^ contents in lenok decreased significantly with increased heat stress exposure. In addition to its role as a coenzyme, NAD^+^ functions as a degradation substrate for enzymes such as poly (ADP ribose) polymerase (PARP) and the sirtuin family of deacetylase enzymes. These enzymes consume NAD^+^ as a substrate to function, and produce nicotinamide (Cerutti et al., 2014; Katsyuba et al., 2020; Chen et al., 2022a). Similarly, NAD^+^ consumption may occur in heat-stressed lenok because nicotinate (nicotinamide amidohydrolase forms nicotinate) accumulated in both S- and T-group fishes. Furthermore, metabolomics revealed precursor substances for DNA (7-methylguanosine, deoxyguanosine, inosine, and guanosine) to change significantly in heat-stressed lenok. Acute heat stress can induce DNA strand breakage and damage in fish (Cheng et al., 2018; Lopes et al., 2021). After DNA damage, NAD^+^ decreased to 20%–30% normal levels by PARPs for DNA repair (Katsyuba et al., 2020). This indicates that DNA damage and repair may occur in heat-stressed lenok, reducing NAD^+^ and leading to a NADH/NAD^+^ imbalance. NADP performs similar redox functions to NAD, and they were more confined to redox protective roles (Ying, 2008). Both NADP^+^ and NADPH in lenok decreased gradually with prolonged heat stress. Decreased NADP^+^ may occur through NAD^+^ depletion, which acts as a parent molecule for the pyridine family of nucleotides including NADP.

The GSH/GSSG redox couple is a most important cellular redox buffer (Schafer and Buettner, 2001). The redox potential of a cell is calculated using the Nernst equation, which is based on the GSH/GSSG ratio (Hamre et al., 2010; Skjærven et al., 2013). The GSH was directly related to scavenging ROS, by which GSH was oxidized into GSSG. The reduced cellular environment occurred in heat-stressed lenok firstly since the GSH and GSSG contents, as well as the GSH/GSSG, increased significantly at 0 h of heat stress. These results were consistent with previous studies wherein an increase in GSH and GSSG levels to eliminate ROS generated in fish liver tissue occurred early during heat stress (Souza et al., 2018). The GSH contents decreased significantly after 12 h of heat stress, while GSSG levels were later comparable to those of control-group fish. These changes indicated that acute heat stress induced a more oxidized conditions in lenok.

Exposure to temperatures beyond those to which a fish is adapted can promote compensatory changes in the metabolic mechanism. After heat stress, metabolomics revealed large amounts of carbohydrates to have been consumed in S- and T-group fish. Carbohydrates are an essential metabolic substratum for maintaining physiological function and survival, especially for stressed fish (Guillen et al., 2019; Chen et al., 2022a). Decreased tissue carbohydrate levels and significant changes in key enzyme activities regulating carbohydrate metabolism guarantee energy demands in heat-stressed fish (Guillen et al., 2019; Yang et al., 2020). Similarly, activity of PK and LDH, the key enzymes for anaerobic glycolysis, were significantly increased at 0 h, reflecting that heat-stressed fish resorted to anaerobic glycolysis to maintain oxygen homeostasis (Currie et al., 2013; Lopes et al., 2021).

Some amino acids (e.g., phenylalanine, tyrosine, glutamate, methionine, and arginine) decreased significantly after heat stress in both S- and T-group fish. Tyrosine and phenylalanine are precursor substances for catecholamine (Mamta et al., 2014). The sympathetic (adrenergic) component of the autonomic nervous system is important for thermal tolerance in fish (Eliason et al., 2011). The stimulatory effect of catecholamines is the protective way in fish species under adverse cellular conditions, especially hypoxemia, which occurred commonly in cold-water fish in heat stress situations (Sandblom and Axelsson, 2011; Gilbert et al., 2019). The tyrosine metabolism pathway was enriched significantly and its catabolites (including Dl-3, 4-dihydroxyphenyl glycol and salidroside) accumulated significantly in both S- and T-group fishes. These results were consistent with previous studies that heat stress induced catabolism of amino acids in fishes (Zhao et al., 2022; Chen et al., 2023). Arginine and glutamate are precursors for creatine, which functions as a cell energy shuttle and is a critical component for maintaining cellular energy homeostasis (Owen and Sunram-Lea, 2011; Prathomya et al., 2017). High levels of creatine, as well as its hydrolysates sarcosine and creatinine observed in S- and T-group fishes, could be metabolic responses to energy stress. As the substrate for nitric oxide (NO) synthetase, arginine can promote the production of NO, which can benefit blood vessel dilation and improve tissue hypoxia state (Haraldsen et al., 2002; Jensen and Agnisola, 2005; Gerber et al., 2017).

The consumption of carbohydrates and amino acids indicated that heat stress induced catabolism in lenok, which led to the accumulations of oxidative products. The redox homeostasis is to maintain a midpoint between oxidation and reduction processes. The NADH/NAD^+^ and NADPH/NADP^+^ ratios increased may because the reduced compounds are growing to contribute to oxidative stress reduction. In addition, NADPH is mainly required for anabolic reactions and cellular oxidative stress defense by glutathione reductase (Ying, 2008; Williams et al., 2020). The activities of HK, PK, NADH dehydrogenase, ALT and AST decreased gradually after 12 h, and dropped to their lowest levels at 48 h. The decreased metabolism might be a compensatory strategy for inhibiting the catabolism, as well as maintaining the redox homeostasis in heat stressed lenok.

The more oxidized conditions may cause oxidative damage in lenok following heat stress. We reported the most apparent changes to be oxidization of phospholipids, the main components of membrane lipids in lenok after heat stress (Li S. et al., 2022; Zhao et al., 2022). Alkyl glycerophosphocholines (e.g., 1-palmitoyl-2-myristoyl-sn-glycero-3-phosphocholine), the alkyl glycerophosphoserines (e.g., 2-oleoyl-1-stearoyl-sn-glycero-3-phosphoserine), PC (16:0e/5, 6-eet), PC (16:1e/17-hdohe), and PC (16:1e/9-hode) increased significantly. While, the unsaturated fatty acyl glycerophosphocholines (e.g., PC 40:8, PC 44:12, Lpc 18:2, and Lpe 18:2) decreased significantly in S- and T-group fishes. These results indicated that fatty acyl in glycerophospholipids was oxidized after heat stress (Serbulea et al., 2017; Anthonymuthu et al., 2018). As the most abundant membrane lipid, changes in glycerophospholipids affect the structure and function of cellular membranes. Additionally, the acylglycerol (e.g., 1, 2-dilinoleoylglycerol) and alkyl phosphatidylglycerol (e.g., 1-palmitoyl-2-oleoyl-phosphatidylglycerol) accumulated in the heat-stressed group. Betaine, derived from choline by an irreversible oxidation reaction in the liver (Kullgren et al., 2013), and sarcosine, an amino acid that forms as an intermediate in the metabolism of choline in the liver, also accumulated. These results indicated that the choline in phospholipids might be oxidized in heat-stressed lenok. Betaine has been reported to effectively prevent liver injury through the inhibition of inflammatory factors and reduction of lipid peroxidation in fish with stressed situation (Prathomya et al., 2017; Chen et al., 2022b).

After 48 h recovery, NAD^+^, NADP^+^ and activities of HK, PK, NADH dehydrogenase, ALT and AST had all recovered, indicating new synthesis. Depletion of carbohydrates was remission after 48 h of recovery, whereas some amino acids (alanine, arginine, valine, glutamate, serine, valine, leucine, methionine and lysine) still occurred at levels lower than in control fishes. Induction of repair mechanisms (i.e., proteasomal degradation and new synthesis) may result in amino acid consumption (Dröge, 2002; Heise et al., 2006). GSH levels were still lower and the more oxidized conditions were not recovered. Excepting the unaltered accumulation of alkyl glycerophosphocholines (e.g., PC 16:0/16:0), alkyl phosphatidylglycerol (e.g., 1-palmitoyl-2-oleoyl-phosphatidylglycerol), PC (16:1e/9-hode), and PC (16:1e/17-hdohe), the lysophosphatidylethanolamine (e.g., lpe18:2), the lysophosphatidylcholine (e.g., 1-pentadecanoyl-sn-glycero-3-phosphocholine), the phosphatidylglycerol (e.g., Pg 42:9), and membrane fatty acid (e.g., arachidic acid) had all accumulated. Similar results were reported for North Sea eelpout that stressful hyperthermia induced hypoxia, and subsequent recovery led to increased oxidative stress, resembling ischemia/reperfusion injury in mammals (Halliwell and Gutteridge, 1999; Heise et al., 2006).

Compared with the S-group, expressions of many amino acids (alanine, arginine, valine, serine, valine, leucine, methionine and lysine) decreased significantly in the T-group fish, indicating that amino acids may play important roles in heat-stress tolerance of lenok. Amino acid catabolism caused elevated ammonia levels, which are neurotoxic and can cause liver damage in heat-stressed lenok. In fish species, glutamate metabolism is primarily delaminated with the production of ammonia derived from amino acid catabolism, which differs from that of mammals (Ballantyne, 2001; Li L. et al., 2022). Significantly increased levels of glutamic acid and glutamine occurred in T-group fish. Additionally, lysine, which can be partially converted into glutamate (Galili et al., 2001; Liu et al., 2019), had strong catabolism. These results are consistent with previous studies that heat stressed fish produced more glutamate and glutamine levels to reduce ammonia toxicity (Liu et al., 2019). Arginine and its catabolites proline and 5-aminopentanoate decreased significantly in S-group fish. As the stable intermediate of the NO synthase (NOS)-catalyzed reaction (Arral et al., 2020), N (omega)-hydroxyarginine accumulated significantly, indicating that the disorders of endogenous NO synthesis derived from arginine in the S group. Limitations in tissue oxygen supply was suggested to lead to loss of equilibrium in fish at the critical thermal maximum (Ern et al., 2020). The vasodilatory action of NO was considered essential to maintain a match between blood flow and tissue oxygen demand in cold water fish (Jensen and Agnisola, 2005; Agnisola and Pellegrino, 2007). NO synthesis disorders may aggravate histohypoxia of S-group lenok, which demonstrated the limited thermal tolerance.

## Data Availability

The datasets presented in this study can be found in online repositories. The names of the repository/repositories and accession number(s) can be found in the article/Supplementary Material.
