# Supplementary material for: Redox state and metabolic responses to severe heat stress in lenok Brachymystax lenok (Salmonidae)
Source: Front Mol Biosci. 2023 May 24;10:1156310. doi: 10.3389/fmolb.2023.1156310 (PMC10244579; doi:10.3389/fmolb.2023.1156310)
Supplement: Supplementary file 1 [file Presentation1.PPTX]

## Slide 1
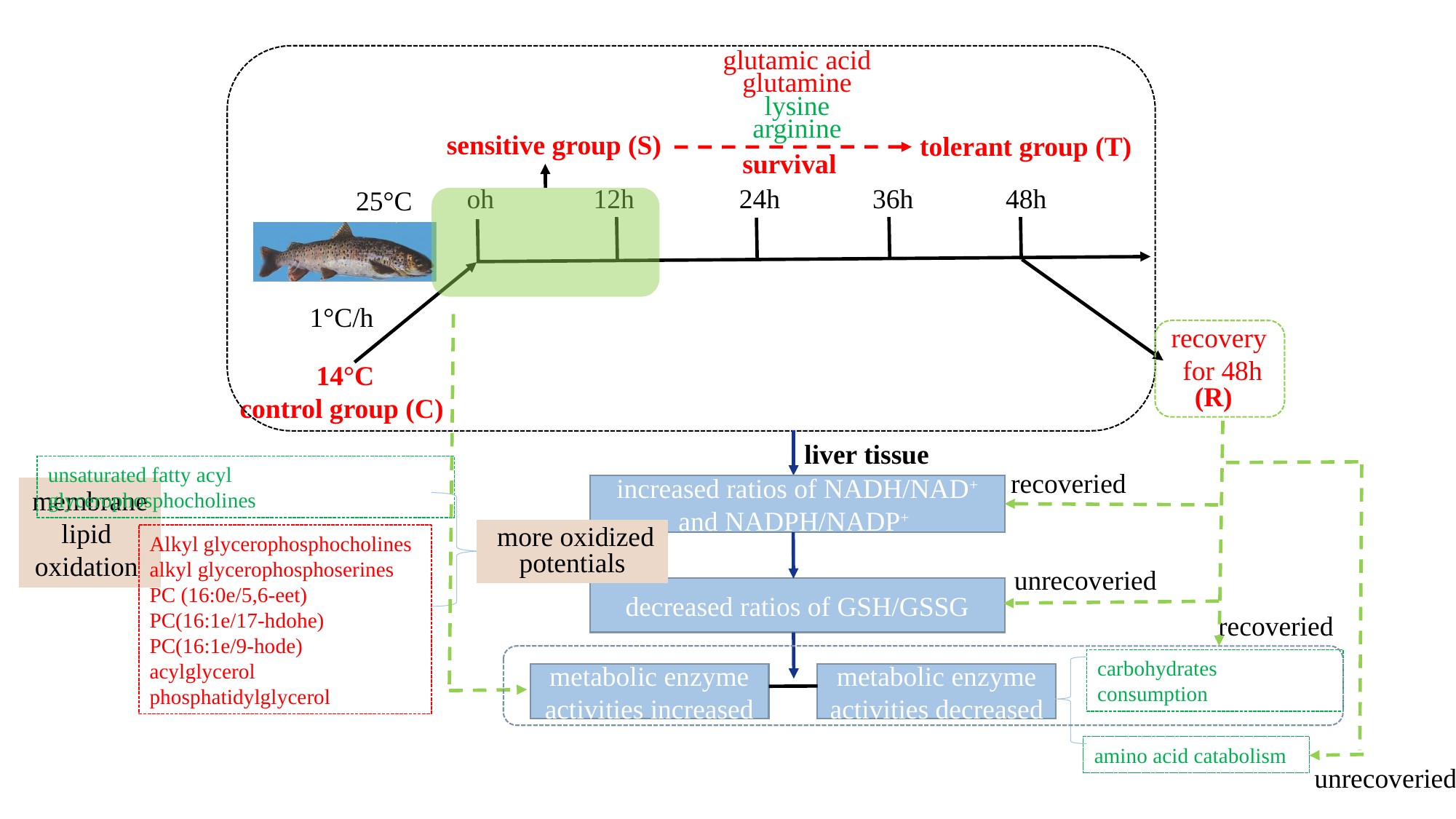

sensitive group (S)
tolerant group (T)
oh
12h
24h
36h
48h
1°C/h
recovery
for 48h
14°C
control group (C)
(R)
25°C
liver tissue
increased ratios of NADH/NAD+ and NADPH/NADP+
unsaturated fatty acyl glycerophosphocholines
recoveried
membrane lipid
oxidation
 more oxidized potentials
Alkyl glycerophosphocholines
alkyl glycerophosphoserines
PC (16:0e/5,6-eet)
PC(16:1e/17-hdohe)
PC(16:1e/9-hode)
acylglycerol
phosphatidylglycerol
unrecoveried
decreased ratios of GSH/GSSG
recoveried
carbohydrates consumption
metabolic enzyme activities increased
metabolic enzyme activities decreased
amino acid catabolism
unrecoveried
glutamic acid
glutamine
lysine
arginine
survival
