# Supplementary material for: Redox state and metabolic responses to severe heat stress in lenok Brachymystax lenok (Salmonidae)
Source: Front Mol Biosci. 2023 May 24;10:1156310. doi: 10.3389/fmolb.2023.1156310 (PMC10244579; doi:10.3389/fmolb.2023.1156310)
Supplement: Supplementary file 3 [file Table6.DOCX]

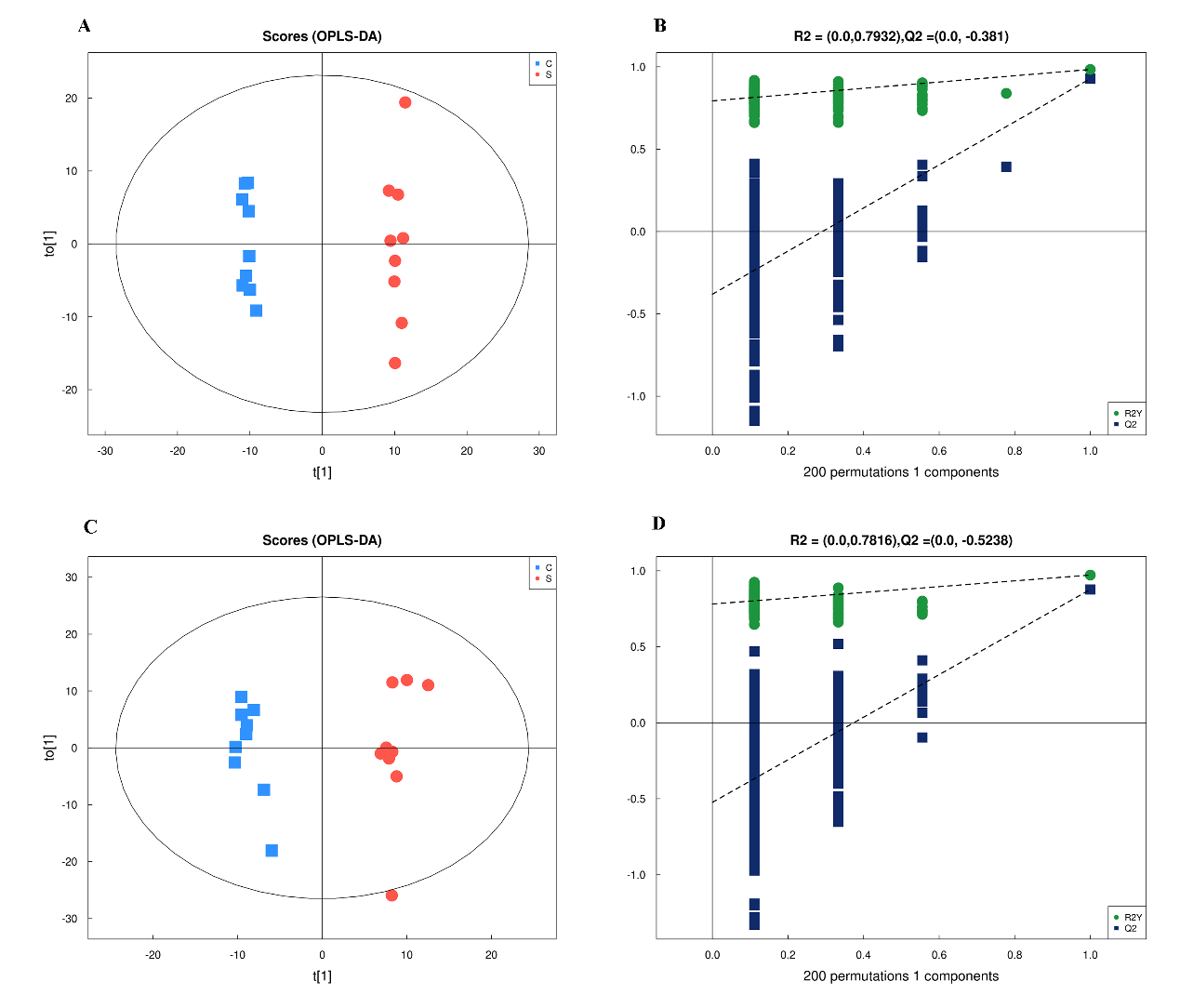


Figure S1 OPLS-DA for discriminating the metabolite profiles of liver in the control (C, blue) and S-group fish (S, green), with each dot representing one liver sample from each treatment in positive mode (A) and negative mode (B); corresponding permutation test in positive mode (C) and negative mode (D).


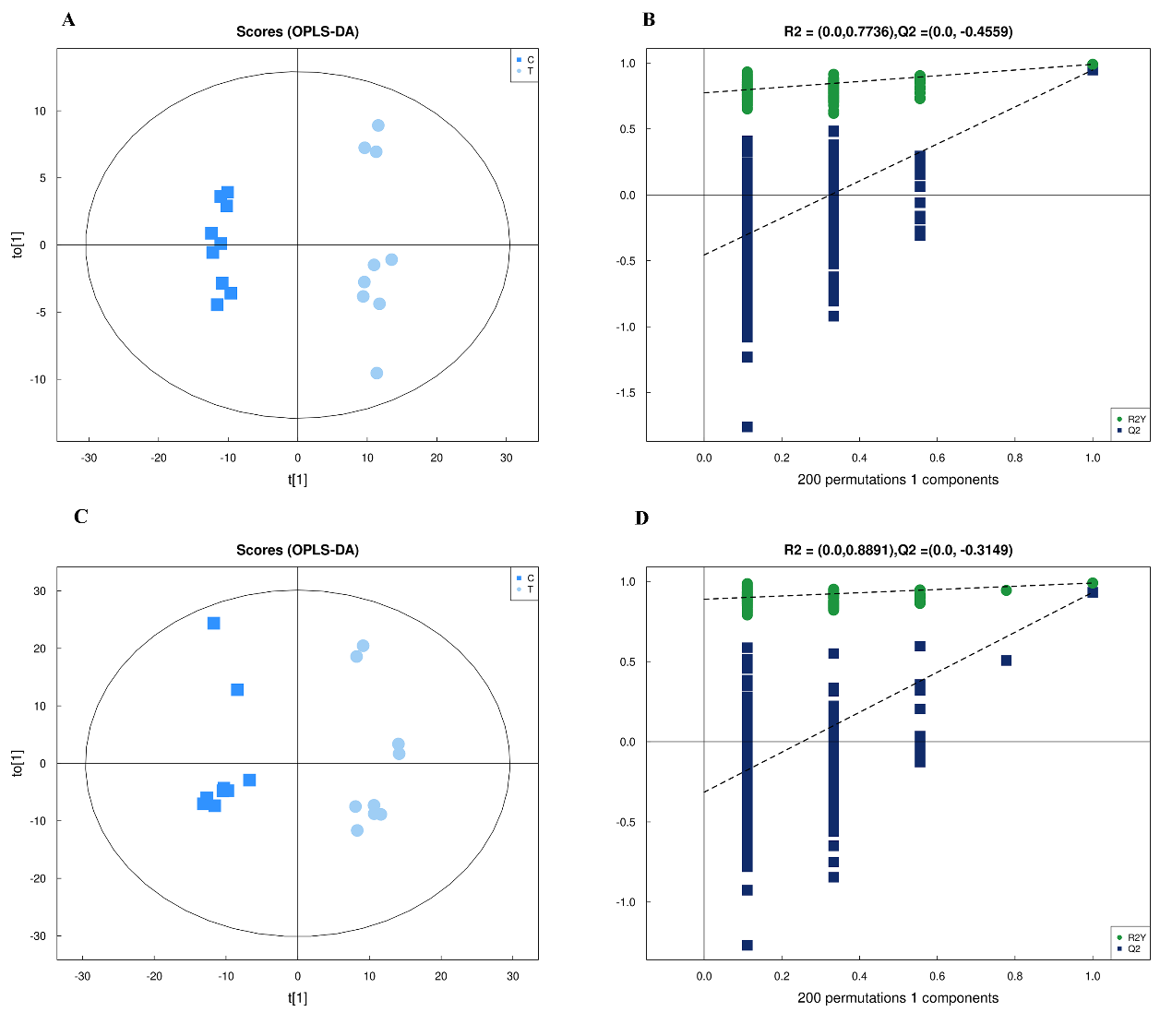


Figure S2 OPLS-DA for discriminating the metabolite profiles of liver in the control (C, blue) and T-group fish (T, green), with each dot representing one liver sample from each treatment in positive mode (A) and negative mode (C); corresponding permutation test in positive mode (B) and negative mode (D).


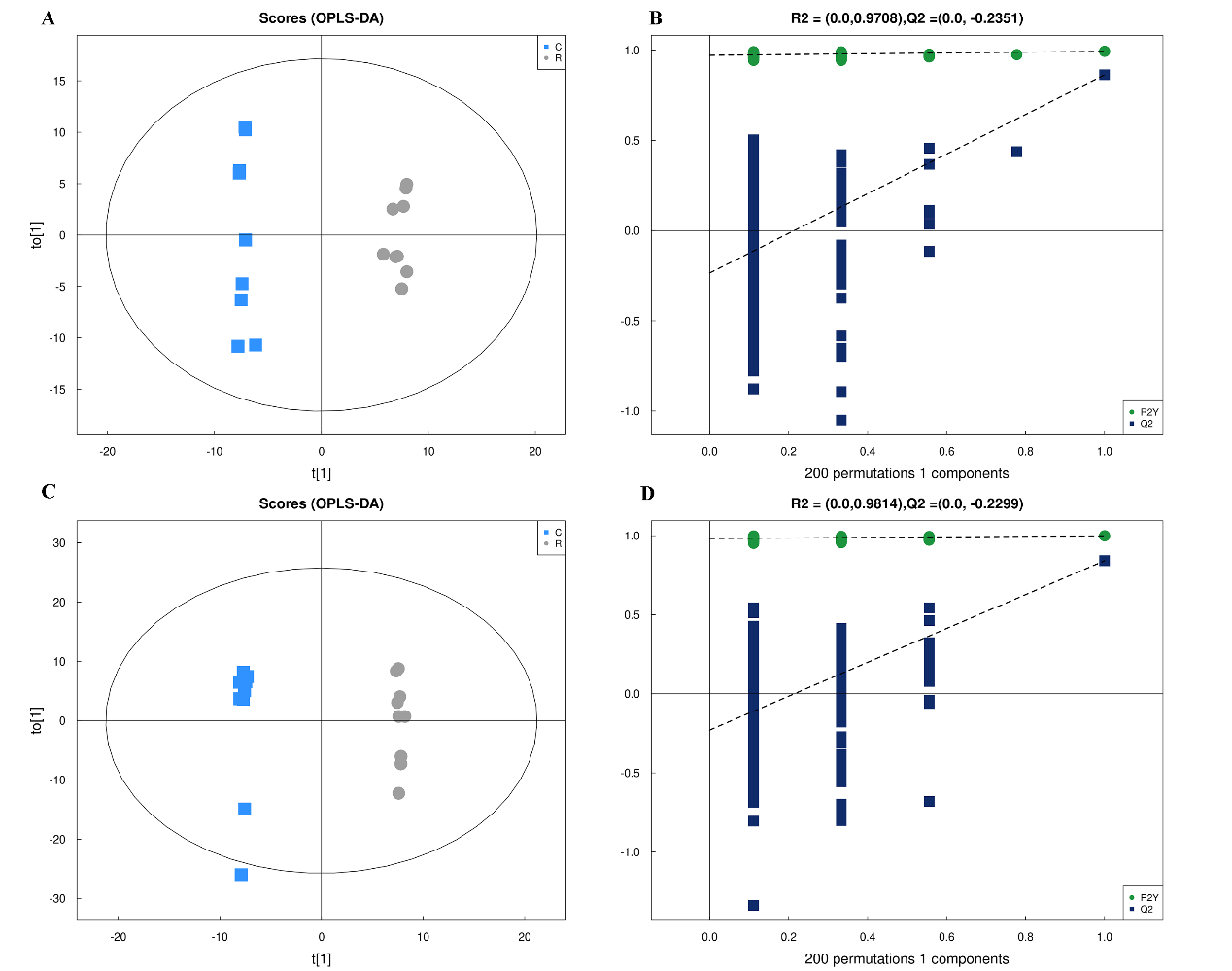


Figure S3 OPLS-DA for discriminating the metabolite profiles of liver in the control (C, blue) and R-group fish (R, green), with each dot representing one liver sample from each treatment in positive mode (A) and negative mode (C); corresponding permutation test in positive mode (B) and negative mode (D).
